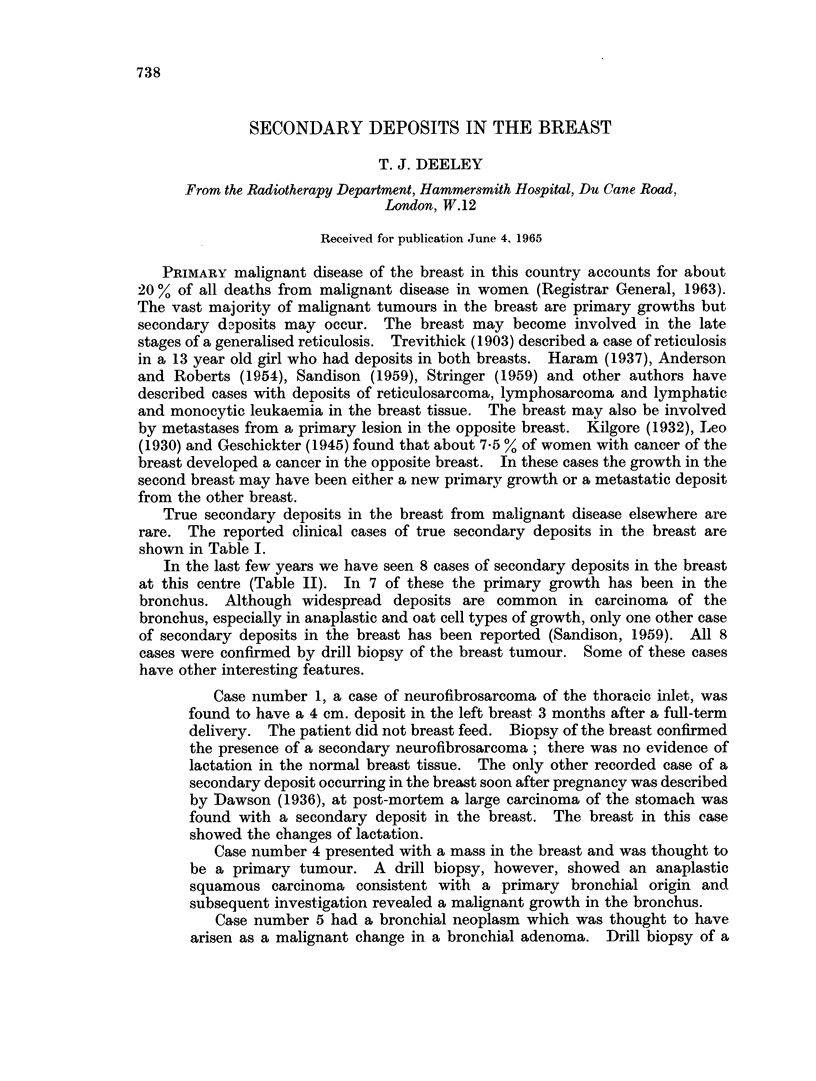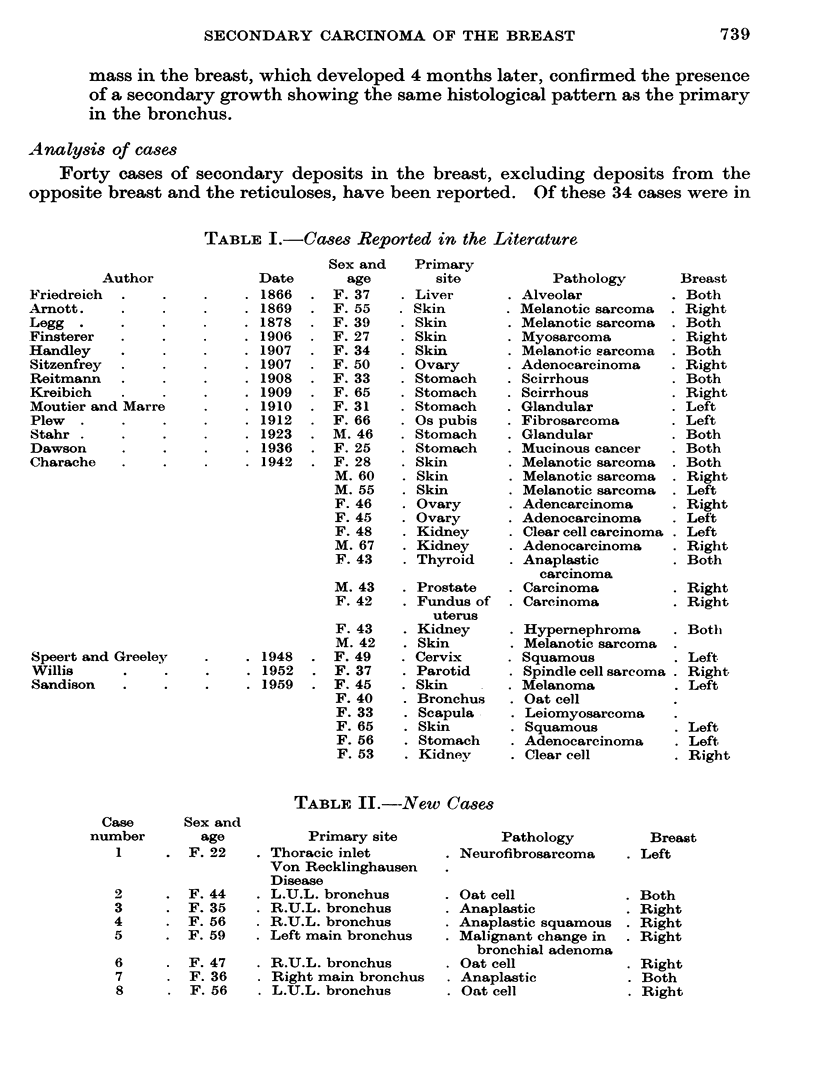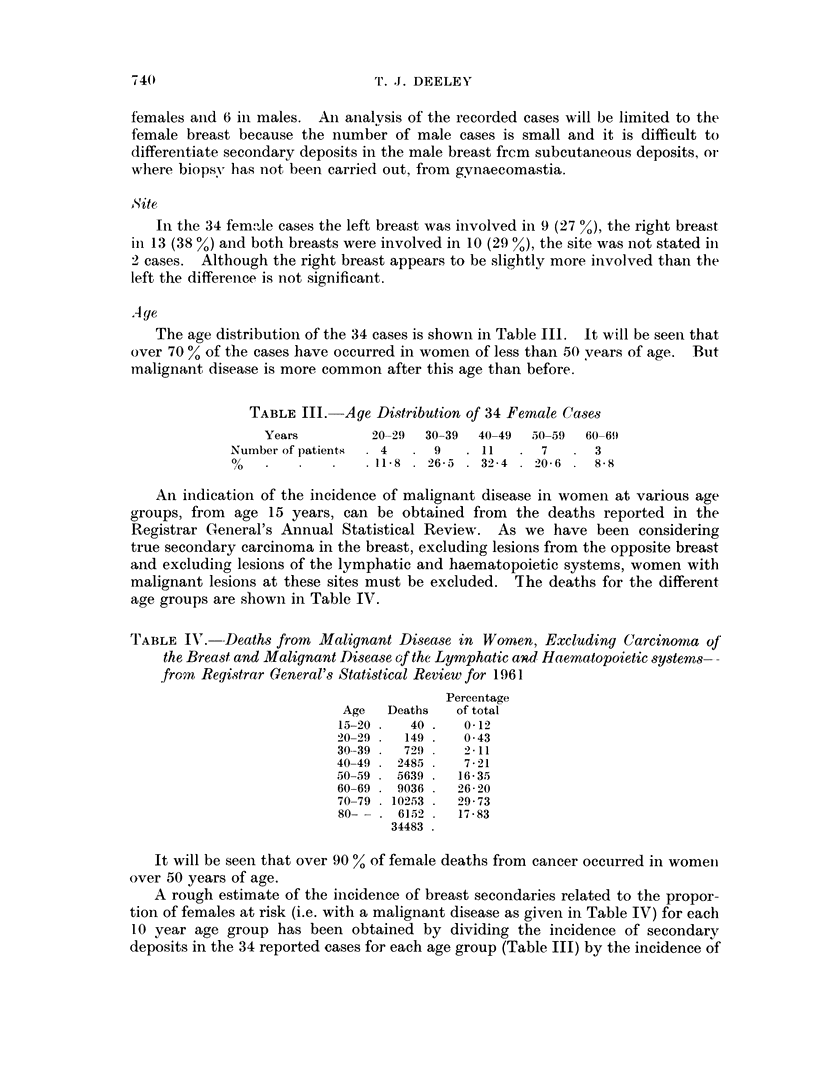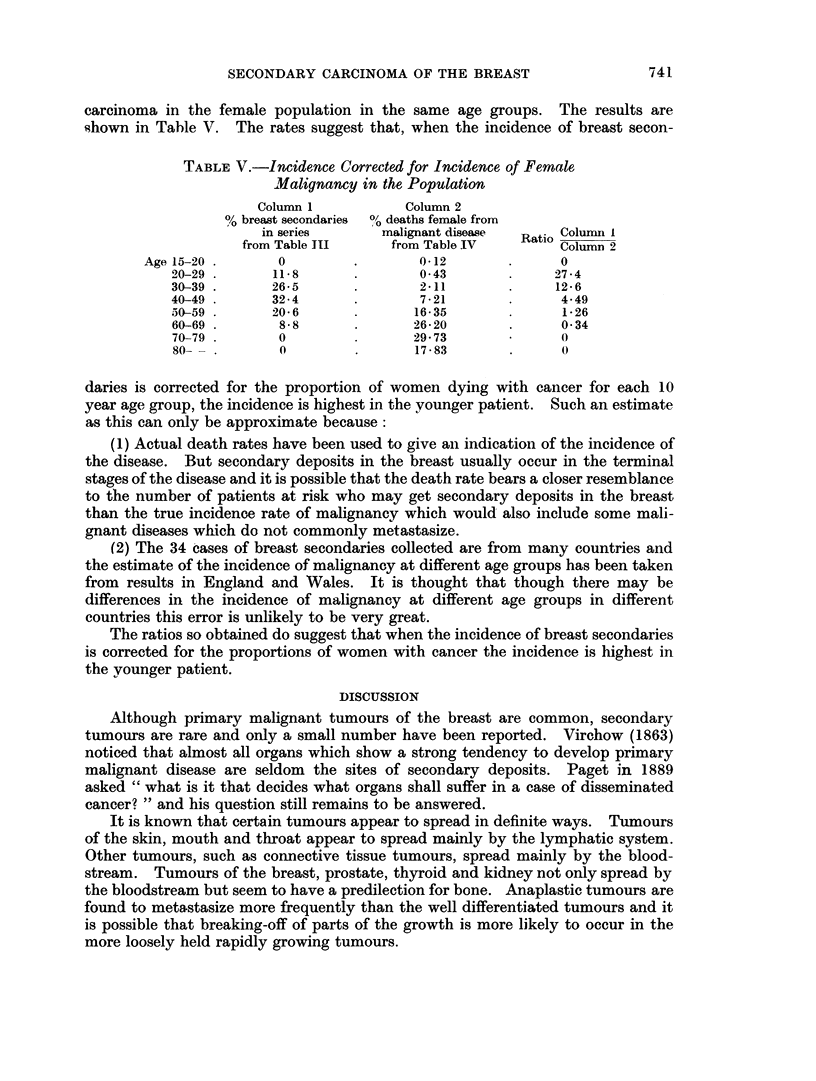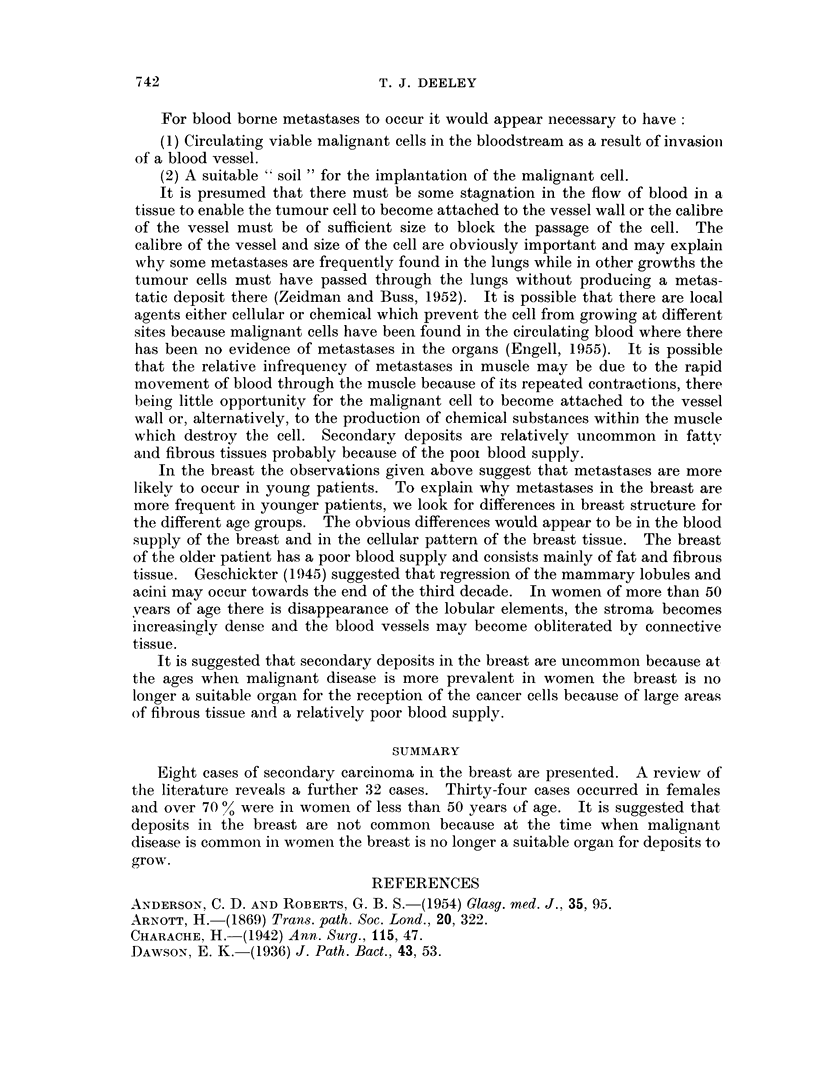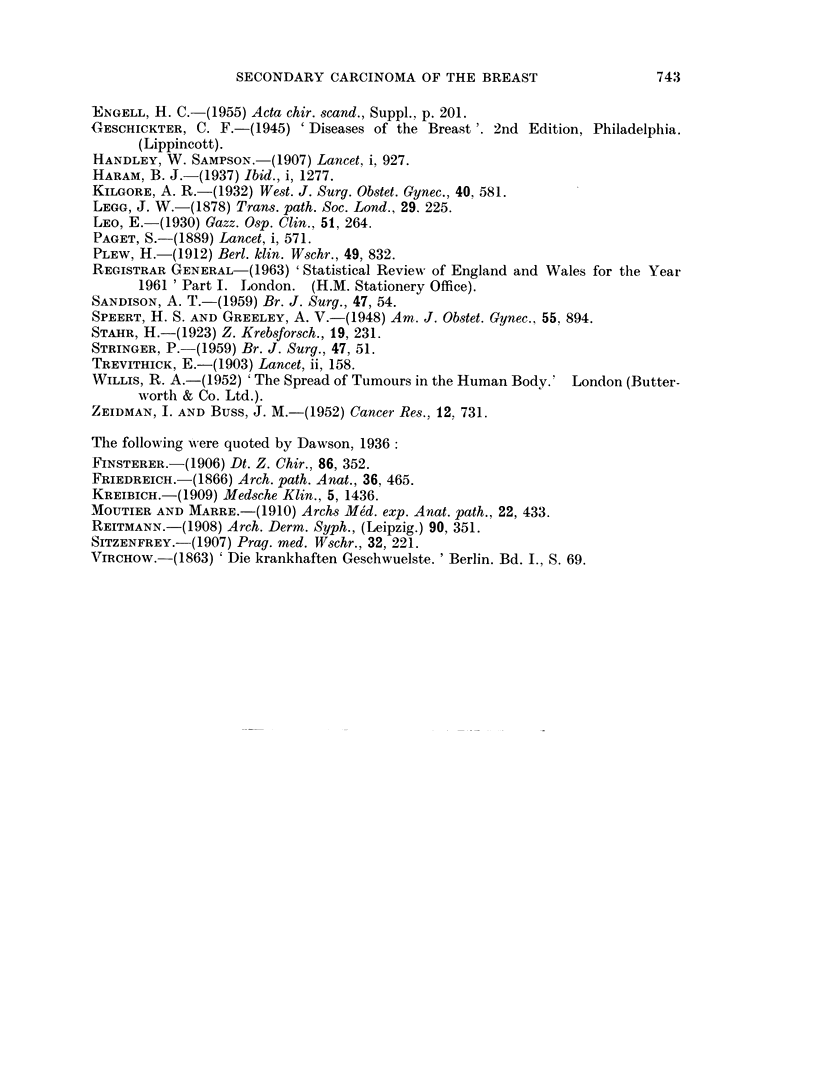# Secondary deposits in the breast.

**DOI:** 10.1038/bjc.1965.85

**Published:** 1965-12

**Authors:** T. J. Deeley


### SECONDARY DEPOSITS IN THE BREAST

T. J. DEELEY

*From the Radiotherapy Department, Hammersmith Hospital, Du Cane Road, London, W.12*

Received for publication June 4, 1965

PRIMARY malignant disease of the breast in this country accounts for about 20% of all deaths from malignant disease in women (Registrar General, 1963). The vast majority of malignant tumours in the breast are primary growths but secondary deposits may occur. The breast may become involved in the late stages of a generalised reticulosis. Trevithick (1903) described a case of reticulosis in a 13 year old girl who had deposits in both breasts. Haram (1937), Anderson and Roberts (1954), Sandison (1959), Stringer (1959) and other authors have described cases with deposits of reticulosarcoma, lymphosarcoma and lymphatic and monocytic leukaemia in the breast tissue. The breast may also be involved by metastases from a primary lesion in the opposite breast. Kilgore (1932), Leo (1930) and Geschickter (1945) found that about 7·5% of women with cancer of the breast developed a cancer in the opposite breast. In these cases the growth in the second breast may have been either a new primary growth or a metastatic deposit from the other breast.

True secondary deposits in the breast from malignant disease elsewhere are rare. The reported clinical cases of true secondary deposits in the breast are shown in Table I.

In the last few years we have seen 8 cases of secondary deposits in the breast at this centre (Table II). In 7 of these the primary growth has been in the bronchus. Although widespread deposits are common in carcinoma of the bronchus, especially in anaplastic and oat cell types of growth, only one other case of secondary deposits in the breast has been reported (Sandison, 1959). All 8 cases were confirmed by drill biopsy of the breast tumour. Some of these cases have other interesting features.

Case number 1, a case of neurofibrosarcoma of the thoracic inlet, was found to have a 4 cm. deposit in the left breast 3 months after a full-term delivery. The patient did not breast feed. Biopsy of the breast confirmed the presence of a secondary neurofibrosarcoma ; there was no evidence of lactation in the normal breast tissue. The only other recorded case of a secondary deposit occurring in the breast soon after pregnancy was described by Dawson (1936), at post-mortem a large carcinoma of the stomach was found with a secondary deposit in the breast. The breast in this case showed the changes of lactation.

Case number 4 presented with a mass in the breast and was thought to be a primary tumour. A drill biopsy, however, showed an anaplastic squamous carcinoma consistent with a primary bronchial origin and subsequent investigation revealed a malignant growth in the bronchus.

Case number 5 had a bronchial neoplasm which was thought to have arisen as a malignant change in a bronchial adenoma. Drill biopsy of a mass in the breast, which developed 4 months later, confirmed the presence of a secondary growth showing the same histological pattern as the primary in the bronchus.

*Analysis of cases*

Forty cases of secondary deposits in the breast, excluding deposits from the opposite breast and the reticuloses, have been reported. Of these 34 cases were in

TABLE I.—*Cases Reported in the Literature*

| Author | Date | Sex and age | Primary site | Pathology | Breast |
|---|---|---|---|---|---|
| Friedreich | 1866 | F. 37 | Liver | Alveolar | Both |
| Arnott | 1869 | F. 55 | Skin | Melanotic sarcoma | Right |
| Legg | 1878 | F. 39 | Skin | Melanotic sarcoma | Both |
| Finsterer | 1906 | F. 27 | Skin | Myosarcoma | Right |
| Handley | 1907 | F. 34 | Skin | Melanotic sarcoma | Both |
| Sitzenfrey | 1907 | F. 50 | Ovary | Adenocarcinoma | Right |
| Reitmann | 1908 | F. 33 | Stomach | Scirrhous | Both |
| Kreibich | 1909 | F. 65 | Stomach | Scirrhous | Right |
| Moutier and Marre | 1910 | F. 31 | Stomach | Glandular | Left |
| Plew | 1912 | F. 66 | Os pubis | Fibrosarcoma | Left |
| Stahr | 1923 | M. 46 | Stomach | Glandular | Both |
| Dawson | 1936 | F. 25 | Stomach | Mucinous cancer | Both |
| Charache | 1942 | F. 28 | Skin | Melanotic sarcoma | Both |
| | | M. 60 | Skin | Melanotic sarcoma | Right |
| | | M. 55 | Skin | Melanotic sarcoma | Left |
| | | F. 46 | Ovary | Adencarcinoma | Right |
| | | F. 45 | Ovary | Adenocarcinoma | Left |
| | | F. 48 | Kidney | Clear cell carcinoma | Left |
| | | M. 67 | Kidney | Adenocarcinoma | Right |
| | | F. 43 | Thyroid | Anaplastic carcinoma | Both |
| | | M. 43 | Prostate | Carcinoma | Right |
| | | F. 42 | Fundus of uterus | Carcinoma | Right |
| | | F. 43 | Kidney | Hypernephroma | Both |
| | | M. 42 | Skin | Melanotic sarcoma | |
| Speert and Greeley | 1948 | F. 49 | Cervix | Squamous | Left |
| Willis | 1952 | F. 37 | Parotid | Spindle cell sarcoma | Right |
| Sandison | 1959 | F. 45 | Skin | Melanoma | Left |
| | | F. 40 | Bronchus | Oat cell | |
| | | F. 33 | Scapula | Leiomyosarcoma | |
| | | F. 65 | Skin | Squamous | Left |
| | | F. 56 | Stomach | Adenocarcinoma | Left |
| | | F. 53 | Kidney | Clear cell | Right |

TABLE II.—*New Cases*

| Case number | Sex and age | Primary site | Pathology | Breast |
|---|---|---|---|---|
| 1 | F. 22 | Thoracic inlet Von Recklinghausen Disease | Neurofibrosarcoma | Left |
| 2 | F. 44 | L.U.L. bronchus | Oat cell | Both |
| 3 | F. 35 | R.U.L. bronchus | Anaplastic | Right |
| 4 | F. 56 | R.U.L. bronchus | Anaplastic squamous | Right |
| 5 | F. 59 | Left main bronchus | Malignant change in bronchial adenoma | Right |
| 6 | F. 47 | R.U.L. bronchus | Oat cell | Right |
| 7 | F. 36 | Right main bronchus | Anaplastic | Both |
| 8 | F. 56 | L.U.L. bronchus | Oat cell | Right |

females and 6 in males. An analysis of the recorded cases will be limited to the female breast because the number of male cases is small and it is difficult to differentiate secondary deposits in the male breast frcm subcutaneous deposits, or where biopsy has not been carried out, from gynaecomastia.

*Site*

In the 34 female cases the left breast was involved in 9 (27 %), the right breast in 13 (38 %) and both breasts were involved in 10 (29 %), the site was not stated in 2 cases. Although the right breast appears to be slightly more involved than the left the difference is not significant.

*Age*

The age distribution of the 34 cases is shown in Table III. It will be seen that over 70 % of the cases have occurred in women of less than 50 years of age. But malignant disease is more common after this age than before.

TABLE III.—*Age Distribution of* 34 *Female Cases*

| Years | 20–29 | 30–39 | 40–49 | 50–59 | 60–69 |
|---|---|---|---|---|---|
| Number of patients | 4 | 9 | 11 | 7 | 3 |
| % | 11·8 | 26·5 | 32·4 | 20·6 | 8·8 |

An indication of the incidence of malignant disease in women at various age groups, from age 15 years, can be obtained from the deaths reported in the Registrar General's Annual Statistical Review. As we have been considering true secondary carcinoma in the breast, excluding lesions from the opposite breast and excluding lesions of the lymphatic and haematopoietic systems, women with malignant lesions at these sites must be excluded. The deaths for the different age groups are shown in Table IV.

TABLE IV.—*Deaths from Malignant Disease in Women, Excluding Carcinoma of the Breast and Malignant Disease of the Lymphatic and Haematopoietic systems—from Registrar General's Statistical Review for* 1961

| Age | Deaths | Percentage of total |
|---|---|---|
| 15–20 | 40 | 0·12 |
| 20–29 | 149 | 0·43 |
| 30–39 | 729 | 2·11 |
| 40–49 | 2485 | 7·21 |
| 50–59 | 5639 | 16·35 |
| 60–69 | 9036 | 26·20 |
| 70–79 | 10253 | 29·73 |
| 80– – | 6152 | 17·83 |
| | 34483 | |

It will be seen that over 90 % of female deaths from cancer occurred in women over 50 years of age.

A rough estimate of the incidence of breast secondaries related to the proportion of females at risk (i.e. with a malignant disease as given in Table IV) for each 10 year age group has been obtained by dividing the incidence of secondary deposits in the 34 reported cases for each age group (Table III) by the incidence of carcinoma in the female population in the same age groups. The results are shown in Table V. The rates suggest that, when the incidence of breast secondaries is corrected for the proportion of women dying with cancer for each 10 year age group, the incidence is highest in the younger patient. Such an estimate as this can only be approximate because :

TABLE V.—*Incidence Corrected for Incidence of Female Malignancy in the Population*

| | Column 1 % breast secondaries in series from Table III | Column 2 % deaths female from malignant disease from Table IV | Ratio Column 1 / Column 2 |
|---|---|---|---|
| Age 15–20 | 0 | 0·12 | 0 |
| 20–29 | 11·8 | 0·43 | 27·4 |
| 30–39 | 26·5 | 2·11 | 12·6 |
| 40–49 | 32·4 | 7·21 | 4·49 |
| 50–59 | 20·6 | 16·35 | 1·26 |
| 60–69 | 8·8 | 26·20 | 0·34 |
| 70–79 | 0 | 29·73 | 0 |
| 80– – | 0 | 17·83 | 0 |

(1) Actual death rates have been used to give an indication of the incidence of the disease. But secondary deposits in the breast usually occur in the terminal stages of the disease and it is possible that the death rate bears a closer resemblance to the number of patients at risk who may get secondary deposits in the breast than the true incidence rate of malignancy which would also include some malignant diseases which do not commonly metastasize.

(2) The 34 cases of breast secondaries collected are from many countries and the estimate of the incidence of malignancy at different age groups has been taken from results in England and Wales. It is thought that though there may be differences in the incidence of malignancy at different age groups in different countries this error is unlikely to be very great.

The ratios so obtained do suggest that when the incidence of breast secondaries is corrected for the proportions of women with cancer the incidence is highest in the younger patient.

### DISCUSSION

Although primary malignant tumours of the breast are common, secondary tumours are rare and only a small number have been reported. Virchow (1863) noticed that almost all organs which show a strong tendency to develop primary malignant disease are seldom the sites of secondary deposits. Paget in 1889 asked " what is it that decides what organs shall suffer in a case of disseminated cancer? " and his question still remains to be answered.

It is known that certain tumours appear to spread in definite ways. Tumours of the skin, mouth and throat appear to spread mainly by the lymphatic system. Other tumours, such as connective tissue tumours, spread mainly by the bloodstream. Tumours of the breast, prostate, thyroid and kidney not only spread by the bloodstream but seem to have a predilection for bone. Anaplastic tumours are found to metastasize more frequently than the well differentiated tumours and it is possible that breaking-off of parts of the growth is more likely to occur in the more loosely held rapidly growing tumours.

For blood borne metastases to occur it would appear necessary to have :

(1) Circulating viable malignant cells in the bloodstream as a result of invasion of a blood vessel.

(2) A suitable " soil " for the implantation of the malignant cell.

It is presumed that there must be some stagnation in the flow of blood in a tissue to enable the tumour cell to become attached to the vessel wall or the calibre of the vessel must be of sufficient size to block the passage of the cell. The calibre of the vessel and size of the cell are obviously important and may explain why some metastases are frequently found in the lungs while in other growths the tumour cells must have passed through the lungs without producing a metastatic deposit there (Zeidman and Buss, 1952). It is possible that there are local agents either cellular or chemical which prevent the cell from growing at different sites because malignant cells have been found in the circulating blood where there has been no evidence of metastases in the organs (Engell, 1955). It is possible that the relative infrequency of metastases in muscle may be due to the rapid movement of blood through the muscle because of its repeated contractions, there being little opportunity for the malignant cell to become attached to the vessel wall or, alternatively, to the production of chemical substances within the muscle which destroy the cell. Secondary deposits are relatively uncommon in fatty and fibrous tissues probably because of the pooı blood supply.

In the breast the observations given above suggest that metastases are more likely to occur in young patients. To explain why metastases in the breast are more frequent in younger patients, we look for differences in breast structure for the different age groups. The obvious differences would appear to be in the blood supply of the breast and in the cellular pattern of the breast tissue. The breast of the older patient has a poor blood supply and consists mainly of fat and fibrous tissue. Geschickter (1945) suggested that regression of the mammary lobules and acini may occur towards the end of the third decade. In women of more than 50 years of age there is disappearance of the lobular elements, the stroma becomes increasingly dense and the blood vessels may become obliterated by connective tissue.

It is suggested that secondary deposits in the breast are uncommon because at the ages when malignant disease is more prevalent in women the breast is no longer a suitable organ for the reception of the cancer cells because of large areas of fibrous tissue and a relatively poor blood supply.

### SUMMARY

Eight cases of secondary carcinoma in the breast are presented. A review of the literature reveals a further 32 cases. Thirty-four cases occurred in females and over 70 % were in women of less than 50 years of age. It is suggested that deposits in the breast are not common because at the time when malignant disease is common in women the breast is no longer a suitable organ for deposits to grow.

### REFERENCES

ANDERSON, C. D. AND ROBERTS, G. B. S.—(1954) *Glasg. med. J.*, **35**, 95.
ARNOTT, H.—(1869) *Trans. path. Soc. Lond.*, **20**, 322.
CHARACHE, H.—(1942) *Ann. Surg.*, **115**, 47.
DAWSON, E. K.—(1936) *J. Path. Bact.*, **43**, 53.

ENGELL, H. C.—(1955) *Acta chir. scand.*, Suppl., p. 201.
GESCHICKTER, C. F.—(1945) 'Diseases of the Breast'. 2nd Edition, Philadelphia. (Lippincott).
HANDLEY, W. SAMPSON.—(1907) *Lancet*, i, 927.
HARAM, B. J.—(1937) *Ibid.*, i, 1277.
KILGORE, A. R.—(1932) *West. J. Surg. Obstet. Gynec.*, **40**, 581.
LEGG, J. W.—(1878) *Trans. path. Soc. Lond.*, **29**. 225.
LEO, E.—(1930) *Gazz. Osp. Clin.*, **51**, 264.
PAGET, S.—(1889) *Lancet*, i, 571.
PLEW, H.—(1912) *Berl. klin. Wschr.*, **49**, 832.
REGISTRAR GENERAL—(1963) 'Statistical Review of England and Wales for the Year 1961' Part I. London. (H.M. Stationery Office).
SANDISON, A. T.—(1959) *Br. J. Surg.*, **47**, 54.
SPEERT, H. S. AND GREELEY, A. V.—(1948) *Am. J. Obstet. Gynec.*, **55**, 894.
STAHR, H.—(1923) *Z. Krebsforsch.*, **19**, 231.
STRINGER, P.—(1959) *Br. J. Surg.*, **47**, 51.
TREVITHICK, E.—(1903) *Lancet*, ii, 158.
WILLIS, R. A.—(1952) 'The Spread of Tumours in the Human Body.' London (Butterworth & Co. Ltd.).
ZEIDMAN, I. AND BUSS, J. M.—(1952) *Cancer Res.*, **12**, 731.

The following were quoted by Dawson, 1936 :

FINSTERER.—(1906) *Dt. Z. Chir.*, **86**, 352.
FRIEDREICH.—(1866) *Arch. path. Anat.*, **36**, 465.
KREIBICH.—(1909) *Medsche Klin.*, **5**, 1436.
MOUTIER AND MARRE.—(1910) *Archs Méd. exp. Anat. path.*, **22**, 433.
REITMANN.—(1908) *Arch. Derm. Syph.*, (Leipzig.) **90**, 351.
SITZENFREY.—(1907) *Prag. med. Wschr.*, **32**, 221.
VIRCHOW.—(1863) 'Die krankhaften Geschwuelste.' Berlin. Bd. I., S. 69.